# Large-Scale Trade in Legally Protected Marine Mollusc Shells from Java and Bali, Indonesia

**DOI:** 10.1371/journal.pone.0140593

**Published:** 2015-12-30

**Authors:** Vincent Nijman, Denise Spaan, K. Anne-Isola Nekaris

**Affiliations:** 1 Oxford Wildlife Trade Research Group, Oxford Brookes University, OX3 0BP, Oxford, United Kingdom; 2 Institute of Neuroethology, University of Veracruz, Xalapa, Mexico; Leibniz Center for Tropical Marine Ecology, GERMANY

## Abstract

**Background:**

Tropical marine molluscs are traded globally. Larger species with slow life histories are under threat from over-exploitation. We report on the trade in protected marine mollusc shells in and from Java and Bali, Indonesia. Since 1987 twelve species of marine molluscs are protected under Indonesian law to shield them from overexploitation. Despite this protection they are traded openly in large volumes.

**Methodology/Principal Findings:**

We collected data on species composition, origins, volumes and prices at two large open markets (2013), collected data from wholesale traders (2013), and compiled seizure data by the Indonesian authorities (2008–2013). All twelve protected species were observed in trade. Smaller species were traded for <USD1.00 whereas prices of larger species were USD15.00–40.00 with clear price-size relationships. Some shells were collected locally in Java and Bali, but the trade involves networks stretching hundreds of kilometres throughout Indonesia. Wholesale traders offer protected marine mollusc shells for the export market by the container or by the metric ton. Data from 20 confiscated shipments show an on-going trade in these molluscs. Over 42,000 shells were seized over a 5-year period, with a retail value of USD700,000 within Indonesia; horned helmet (*Cassis cornuta*) (>32,000 shells valued at USD500,000), chambered nautilus (*Nautilus pompilius*) (>3,000 shells, USD60,000) and giant clams (*Tridacna* spp.) (>2,000 shells, USD45,000) were traded in largest volumes. Two-thirds of this trade was destined for international markets, including in the USA and Asia-Pacific region.

**Conclusions/Significance:**

We demonstrated that the trade in protected marine mollusc shells in Indonesia is not controlled nor monitored, that it involves large volumes, and that networks of shell collectors, traders, middlemen and exporters span the globe. This impedes protection of these species on the ground and calls into question the effectiveness of protected species management in Indonesia; solutions are unlikely to be found only in Indonesia and must involve the cooperation of importing countries.

## Introduction

Tropical marine molluscs have lost much of their historical usage as medicine, tools or religious symbols [1] but their economic value as a source of protein [2] and as ornaments or decorations is possibly greater now than ever before [3]. Ornamental shells are still traded in large quantities [4] [5] [6] and perhaps even more so today because of the rise of trade via the Internet [3]. Throughout the world, gastropod, cephalopods and bivalve shells are bought or collected as ‘portable memories’ when people are vacationing [3] [7]. Tropical shells, with their attractive colours and hues, various morphologies with intricate spikes and curves, are especially popular souvenirs. The development of international and local tourism in the tropics over the last decade has increased the demand and trade in souvenirs, with consequent pressures on marine resources [8].

Indonesia, the world’s largest archipelago, is particularly rich in marine molluscs, and several species have been, or still are, traded in large numbers [9] [10]. Larger species, especially, have suffered negatively from (over)exploitation [9]. In an attempt to reduce the impact of collectors, in 1987 twelve species were added to Indonesia’s protected species list. These are: bear paw clam *Hippopus hippopus*, China clam *H*. *porcellanus*, saffron-coloured giant clam *Tridacna crocea*, southern giant clam *T*. *derasa*, giant clam *T*. *gigas*, small giant clam *T*. *maxima*, fluted giant clam *T*. *squamosa*, Triton’s trumpet *Charonia tritonis*, horned helmet *Cassis cornuta*, commercial top shell *Trochus niloticus*, marbled turban *Turbo marmoratus* and chambered nautilus *Nautilus pompilius*. Some of these, such as the marbled turban and commercial top shell are relatively small and are collected and exported in large quantities for the ornamental shell trade. Several clam species, including all species of giant clam *Tridacna* spp, are used for food and for floor tiles but also as decoration or trays, and Triton’s trumpet, horned helmet, and chambered nautilus have large and beautiful shells that are used as ornaments [9] [11].

We here aim to (a) present data on the trade in protected marine mollusc shells in two of Indonesia’s largest open markets, Pangandaran and Pasir Putih, both on the island of Java, (b) explore the relationships between sizes and prices of the shells observed in trade, (c) report on the existence of wholesale traders that export large quantities of protected marine molluscs, and (d) give an overview of the trade in these shells from Indonesia though a compilation of confiscation data as reported by the Indonesian authorities and media. We start, however, with an overview of the legal protection and management of marine molluscs in Indonesia.

## Legal Protection and Management of Marine Molluscs in Indonesia

The 12 marine molluscs listed above were first added to the list of protected species through a decree from the Minister of Forestry on 12 January 1987 (Surat Keputusan Menteri Kehutanan No 12/Kpts/II/1987). Later this was consolidated into Act of the Republic of Indonesia No. 5 of 10 August 1990 concerning the Conservation of Living Resources and their Ecosystems (Undang-undang Republik Indonesia No 5 tahun 1990) and Regulation 7 concerning the Preservation of Plants and Animals of 27 January 1999 (Peraturan Pemerintah No 7 tahun 1999). Act No 5 states that: “Any and all persons are prohibited to (a) Catch, transport, and trade in a protected animal in a live condition; (b) Keep, possess, transport, and trade in a protected animal in a dead condition; (c) Transfer a protected animal from one place to another, within or outside Indonesia (d) Trade, keep or possess bodies, or other parts of a protected animal or the goods made of parts of the animal, or transfer from one place in Indonesia to another, within or outside Indonesia”. Penalties that can be imposed when these laws are broken can total fines of up to IDR 100,000,000 (~USD 10,000) and imprisonment for up to five years.

Indonesia acceded to the Convention on International Trade of Endangered Species of Wild Fauna and Flora (CITES) in December 1978, which entered into force in March 1979 [12]. Species regulated by the Convention are listed in three Appendices. Those listed in Appendix I are prohibited from commercial international trade, while those in Appendix II or III are permitted in international trade, providing trade is carried out in accordance with the requirements of CITES and national legislation. A small number of marine molluscs are included on the Appendices of CITES. Of these only the giant clams, listed on Appendix II, occur in Indonesian waters, and Indonesia only allows export of maricultured giant clams (these are exported as captive-born, code F, under CITES terminology). Proposals have been made by Australia for Triton’s trumpet to be included on Appendix II and currently data are collected to evaluate whether a CITES II listing is appropriate for chambered nautilus, and indeed other related nautilus species (see Discussion).

Indonesia’s legislation stipulates that trade in all non-protected species, native to Indonesia, whether listed on CITES or not, be regulated by a harvest and export quota system. The quotas are set on an annual basis for all species at a meeting of various stakeholders including the Directorate General of Forest Protection and Nature Conservation (Perlindungan Hutan dan Konservasi Alam or PHKA, also acting as the CITES Management Authority), the Indonesian Institute of Sciences (Lembaga Ilmu Pengetahuan Indonesia or LIPI, the CITES Scientific Authority), non-government organizations and traders. The basis for the quotas are requests submitted by the regional offices for the Natural Resources Conservation Agency (Balai Konservasi Sumber Daya Alam or BKSDA) as well as requests from traders, to the PHKA. Quota-setting is meant to be based on results of non-detriment finding surveys for all traded species, to determine sustainable harvest levels for any given species or population in any given area. However, in Indonesia non-detriment findings are normally not carried out in any systematic fashion [12] [13], and to date, it is understood that there have been no non-detriment findings carried out for any species of mollusc. Consequently, harvest quotas are essentially based on the requests submitted by the BKSDA and divided by province or district. While the above regulations should be irrelevant for protected species as no trade in them is allowed, an exception has been made for commercial top shell. On 2 June 1999 a decree from the Minister of Forestry (Keputusan MenHut 385/kpts-II/1999) designated commercial top shell as a species that could be harvested, despite its protected status, following a set of regulations and a quota setting process similar if not identical to that outlined above for non-protected species. In addition, a minimum collection size of 80 mm should be adhered to and quotas for commercial top shell are typically in the order of 200 metric tons (equivalent to some 2 million individual shells) per year (to the best of our knowledge these are still in place). These quotas are mostly allocated to the central and eastern provinces of South and Central Sulawesi, the Moluccas and West Papua, with no collection permitted in western Indonesia (viz. in the seas surrounding Sumatra, Java and Indonesian Borneo). It is unclear to what extent the trade in commercial top shell is on-going, but data from Arifin et al. [14] and Barhunuddin [15] demonstrate that in the period after inclusion of the species on the protected species list but prior to permitting harvest, and thus at a time when no trade was allowed, on average close to 30 metric tons were traded annually from the Moluccas and the Lesser Sunda Islands. In the early 1990s a small number of hatcheries–partially as research projects by Indonesian universities- were established for the production of commercial top shell [16] but it is unclear if these facilities are still active, and if so how many shells they produce.

## Results

### 1. Numbers and species composition in Pangandaran and Pasir Putih

**Fig 1:**
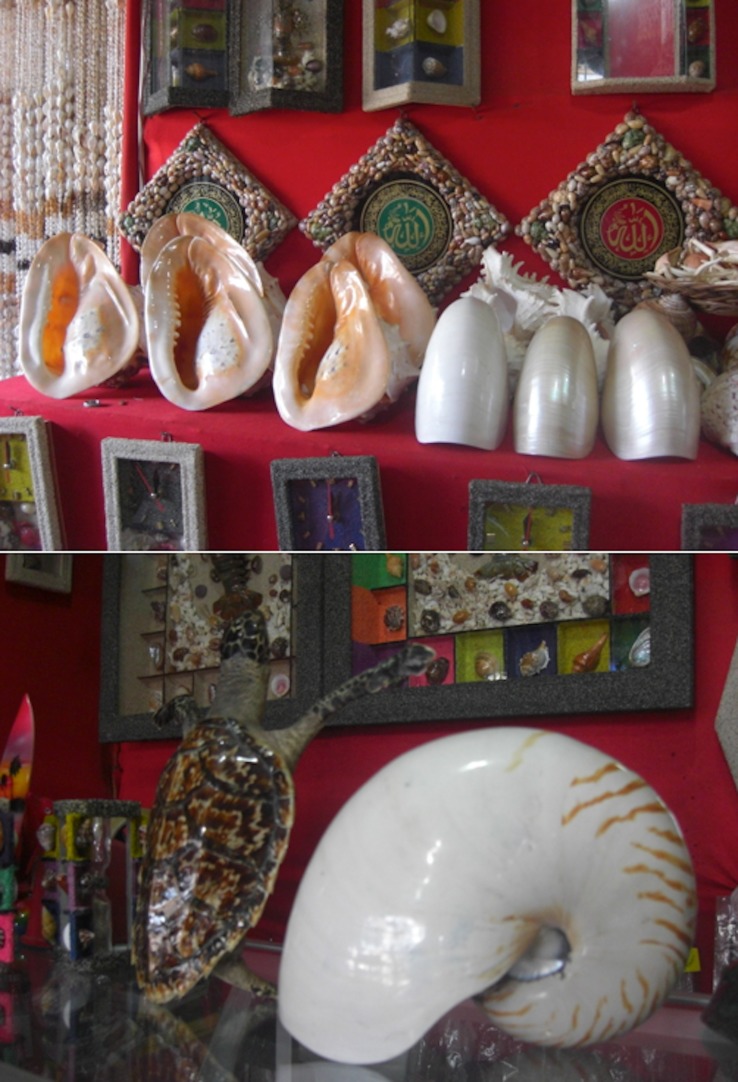
Legally protected shells for sale. Protected marine mollusc shells for sale in Pangandaran, Indonesia, June 2013. Top: Horned helmet *Cassis cornuta* and chambered nautilus *Nautilus pompilius*. Bottom: Chambered nautilus

We observed a large range of protected marine species traded as curios. In Pangandaran, of the estimated 240 and 260 shops that sold marine curios (January and June 2013, respectively), around 80 to 90 sold at least one species of protected shell, and often many more. The shops with the largest variety of protected wildlife (typically 8 to 9 species of the listed 12) were situated in the tourism market at the northern end of the village. For Pasir Putih in June 2013, of the estimated 150 shops that sold marine curios, at least 45 offered legally protected shells for sale (Fig 1). These shops were equally spread out over the entire beach front, with three shops situated on the main road dissecting the village.

Traded species included bear paw clam (100s), China clam (100s), marbled turban (10s), and commercial top shell (1000s). Chambered nautilus, and giant clams were traded openly in both locations, as were the giant shells of horned helmet, Triton’s trumpet and false trumpet *Syrinx aruanus*, with three specimens of crusty nautilus *Allonautilus scrobiculatus* observed in one shop in Pasir Putih (Table 1). All but the latter two species are protected under Indonesian law. These species were offered openly for sale, clear for all to see, and because of their size they were often prominently displayed. Apart from one trader in Pasir Putih, when discussing nautilus shells, none of the traders mentioned the protected status of these species and the illegality of the trade. Qualitatively, the trade in protected species did not differ from that of unprotected species.

**Table 1:** Protected shells for sale in Java. Trade in marine shells at Pangandaran and Pasir Putih beach resorts, Java, Indonesia, in January and June 2013, with between brackets the number of stalls where the species was offered for sale. Status: protected = protected under Indonesian law since 1987, Appendix II = included on Appendix II of the Convention on International Trade in Endangered Species of Fauna and Flora (CITES) since 1983, regulating all international trade.

Species	Status	Pangandaran 5–6 January	Pangandaran 8–10 June	Pasir Putih 30 June
Chambered nautilus *Nautilus pompilius*	protected	56 (12)	42 (12)	70 (16)
Crusty nautilus *Allonautilus scrobiculatus*	not protected			3 (1)
Horned helmet *Cassis cornuta*	protected	111 (20)	89 (23)	130 (17)
Triton’s trumpet *Charonia tritonis*	protected	15 (5)	17 (7)	8 (4)
False trumpet *Syrinx aruanus*	not protected	27 (4)	11 (3)	3 (2)
Giant clam *Tridacna* spp	protected, Appendix II	25 (12)	39 (20)	35 (11)

There was a small but statistically significant temporal difference in the relative numbers of each of the different species offered for sale at Pangandaran (X^2^ = 11.42, df = 4, P = 0.02). Compared to all the other species combined, in June, significantly more giant clams were on sale than in January (X^2^ = 6.90, df = 1, P = 0.01). There was no temporal difference in the number of shops that offered each of the species for sale (X^2^ = 1.23, df = 4, P = 0.85). At a spatial level comparing the surveys conducted in June 2013, the species composition and numbers differed between Pangandaran and Pasir Putih (X^2^ = 17.72, df = 4, P = 0.01). Relative to all the other species combined, there were significantly more false trumpet and Triton’s trumpet for sale in Pangandaran than Pasir Putih (X^2^ = 23.10, df = 1, P<0.01 and X^2^ = 23.23, df = 1, P<0.01 for false trumpet and Triton’s trumpet, respectively). The average number of shells of each species sold in individual shops did not differ between Pangandaran and Pasir Putih (X^2^ = 2.68, df = 3, P = 0.40, test done on numbers per 4 shops and giant clam excluded as to avoid too many low expected values): for most species between 1 and 4 individual shells were on offer per shop and only horned helmets were more abundant at Pasir Putih with an average of almost 8 per shop.

**Fig 2:**
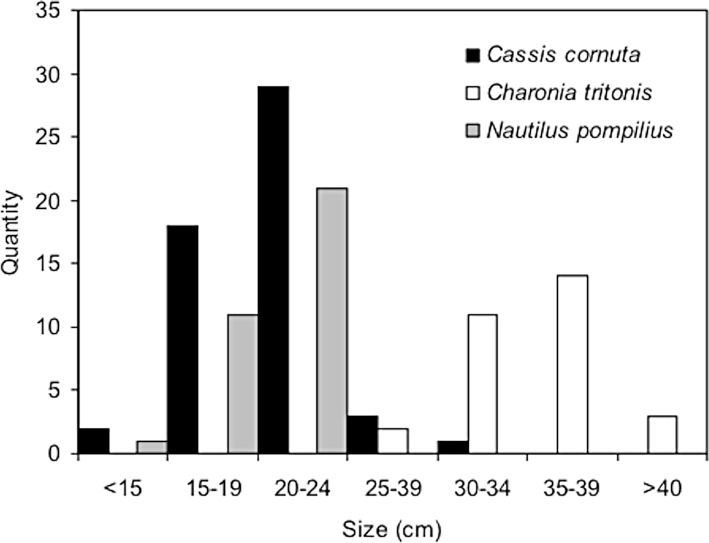
Size classes of shells in trade. Distribution of sizes of three commonly traded protected marine mollusc shells in Pangandaran, Indonesia.

For three species enough data were collected from Pangandaran to evaluate the different size classes offered for sale (i.e., chambered nautilus (n = 33), horned helmet (n = 53) and Triton’s trumpet (n = 30) (Fig 2)). Chambered nautilus ranged in size from 14 to 22 cm, with most of the specimens in the 16–18 cm size class; the largest ones are close to the maximum size the species attains. Horned helmet ranged in size from 14 to 23 cm, with a peak in the 20–24 cm size class. None were close to the maximum size of around 31 cm. Triton’s trumpet ranged between 28 and 41 cm with the majority in the 30–34 cm and 35–39 cm size classes. The largest Triton’s trumpets we observed in Panagandaran are close to the maximum size the species attains.

### 2. Origins and clientele

The traders in Pangandaran were unanimous in stating the origin of the nautilus and large shells as either Madura (an island off the east coast of Java) or from Madurese people (who besides on Madura reside in appreciable numbers along the north coast of East Java; this includes the region where Pasir Putih is situated); one trader however stated coming from the area around Jepara, a town in northeastern Central Java. The deep Indian Ocean off Pangandaran was said not to contain any of these shells (or if there were they were not collectable). Traders in Pasir Putih stated “nautilus and other shells originated from the sea there” (i.e. the Java Sea). The species were said to be collected from shallow areas, thus facilitating their collection, but this probably does not refer to the nautilus. The smaller species were said to be collected in the seas off Pangandaran and Pasir Putih, respectively.

We conducted our study during weekends (that coincided with public holidays) to maximise the number of shops that we were available to survey. According to staff at the entry gate, Pangandaran receives an estimated 2 million visitors a year. Given that the nearest cities are several hours away a large proportion would spend at least one night in the area. This would suggest that, on average, some 5000–10,000 people are present on any given day, and more on weekends. According to ticket sellers at the gate at least 95% of visitors are Indonesian. We have no data on annual visitor numbers for Pasir Putih but during the 10 days of Lebaran holiday at the beginning of August 2013, some 25,000 visitors went through the entry gates [17]. On all days visited, both in Pangandaran and Pasir Putih 1,000s of holidaymakers and tourists were present, the vast majority of them Indonesian. These observations were corroborated by our findings, with shop owners indicating that the main clientele is unequivocally Indonesian, originating mostly from Jakarta and Bandung in the case of Pangandaran, and Jakarta and Surabaya for Pasir Putih. During the three visits only three times did we see non-Asian tourists, twice in Pangandaran and once in Pasir Putih. Evidently, both Pangandaran and Pasir Putih cater predominantly to Indonesian tourists.

### 3. Whole-sale trade from Java

Two wholesale companies were operational in Pangandaran. Tourists visiting their shops could buy individual items as in any other shop, but these wholesalers additionally catered for the export market. Species offered for sale included chambered nautilus, horned helmet, Triton’s trumpet, and false trumpets. One of the wholesalers informed us that he exported his merchandise to Malaysia and Saudi Arabia on a bimonthly basis; the other wholesaler did not specify his shipments’ destinations. We found five additional Indonesian wholesale companies that offered protected marine molluscs for sale through their company websites, details of which we keep confidential. Two were based in East Java (Surabaya and Malang), two in Bali (Kutai and Denpasar) and one in Sulawesi (Toli-Toli). They mostly offered nautilus shells, Triton’s trumpet and horned helmet for sale, with two of them also stocking false trumpets. Traders place orders directly to the wholesalers, often providing details of their own trading company. The wholesale company in Sulawesi had a minimum order size of 1 metric ton, whereas another in Java made it clear that it was able to supply one container per month.

### 4. Assessment of illegal trade in marine molluscs in and from Java and Bali

We found reports of 20 separate cases pertaining to the illegal trade of protected marine molluscs in the period 2005–2013 (Table 2). In total over 42,000 shells of protected marine molluscs were seized by the authorities. The majority of these were horned helmet (>32,000), chambered nautilus (>3,000) and giant clams (>2000). At least two-thirds of these were intended for international trade with the largest volumes destined for China and the USA. Two cases involved shipments from the island of Enggano and the nearby town of Bengkulu on Sumatra, and one from the island of Sulawesi; all had trade links to Java. Below is a summary of these 20 cases.

**Table 2:** Confiscated marine shells. Large-scale seizures (>25 individuals) of three species of large marine molluscs in western Indonesia (see text for details).

Date	Location	Chambered nautilus *Nautilus pompilius*	Horned helmet *Cassis cornuta*	Triton’s trumpet *Charonia tritonis*	Ref.
Aug-Dec 2005	Denpasar, Bali	33	106	61	19
May-Sep 2008	Denpasar, Bali	27	58	6	21
Jan 2009	Kendari, Sulawesi		7,000		24
Sep 2009	Surabaya, Java	558	2959	56	30
Dec 2010	Jakarta, Java	20	4	2	31
Jul 2011	Sumenep, Madura		30	11	32
Jun 2012	Surabaya, Java	485	20,515	204	33

In August 2005, 500 commercial topshell and 20 horned helmet shells were confiscated in the city of Bengkulu, Sumatra that had been collected in the waters off the island of Enggano [18]. The news report made it clear that the horned helmets were confiscated because they were protected species, but stated that the topshells were not protected and were confiscated because of the absence of collection permits. In four different raids in August and December 2005 and June 2006, the Bali regional Natural Resource Management Office confiscated 19 bear paw clam, 5 marbled turban, 105 commercial topshell, 106 horned helmet, 61 Triton’s trumpet, 95 giant clam, and 33 chambered nautilus [19]. In November 2007, 480 large boxes with over 5,000 shells, including horned helmet and chambered nautilus, were confiscated in Surabaya, en route to the USA [20]. Between May and September 2008, in four raids in Bali, 58 horned helmet, 6 Triton’s trumpet, 5 giant clam, and 27 chambered nautilus were confiscated [21]. In January 2008 a container with an unknown quantity of horned helmet and Triton’s trumpet were confiscated from a ship bound for Surabaya, in Kendari, Sulawesi: allegedly, the shells had been collected in Central Sulawesi [22]. In September 2008, hundreds of shells of Triton's trumpet and nautilus were confiscated at Denpasar International Airport, on their way from Bali to New Caledonia [23]. In January 2009, the Sulawesi regional Natural Resource Management Office confiscated 7,000 horned helmet shells in Kendari, Central Sulawesi en route to the city of Surabaya. They were purchased from local fishermen from North Sulawesi and the island of Banggai, for USD 0.25–0.50 per shell [24]. Another report of the same raid reported the additional presence of large numbers of Triton’s trumpet [25]. In March 2009, two people were arrested in Banyuwangi in possession of 200 giant clam collected from the Bali Strait and intended for exported to Vietnam [26]. A month later, in the same city, 250 commercial topshell were confiscated from fishermen from Sitobondo (the regency Pasir Putih is situated in). They were caught in the waters off Madura and were on their way to Sitobondo, and presumably Pasir Putih [27]. In May 2009, 5 horned helmet shells were confiscated in Bali [28] and in September 2009, 200 chambered nautilus were seized at Denpasar international airport [29]. In September 2009, 2959 horned helmet, 56 Triton’s trumpet and 558 chambered nautilus were confiscated in Surabaya harbour; the cargo was destined for China [30]. In December 2010, 4 horned helmet, 6 commercial top shell, 2 Triton’s trumpet and 20 chambered nautilus were confiscated in a raid in Jakarta [31]. In July 2011 two people were arrested for possession and transport of 30 horned helmet and 11 Triton’s trumpet in the city of Sumenep, opposite Surabaya, on the island of Madura. Allegedly they bought the shells for USD 0.40 (horned helmet) and USD 1.00 (Triton’s trumpet) from local fishermen and sold them on for USD 0.60 and USD 1.80, respectively [32]. In June 2012, two containers with 20,515 horned helmet, 204 Triton’s trumpet, 485 chambered nautilus, and 768 false trumpet shells, bound for China, were confiscated in Surabaya [33]. Finally, in June 2013 it was reported that a company from Bogor, West Java, had collected 200 tonnes of giant clam from the reefs off Enggano Island, with the clam under wraps in the village of Kahyapu, Enggano awaiting permits to be transported to West Java [34].

Over the period 2005–2013 a total 75 giant clams and 34 bear paw clams were exported as shells and confiscated in Austria (5 giant clams), Czech Republic (7 giant clams, 8 bear paw clams), Slovenia (16 giant clams, 16 bear paw clams), and the USA (47 giant clams, 10 bear paw clams). In addition, the USA allowed the import of 2 wild-caught giant clamshells from Indonesia.

**Fig 3:**
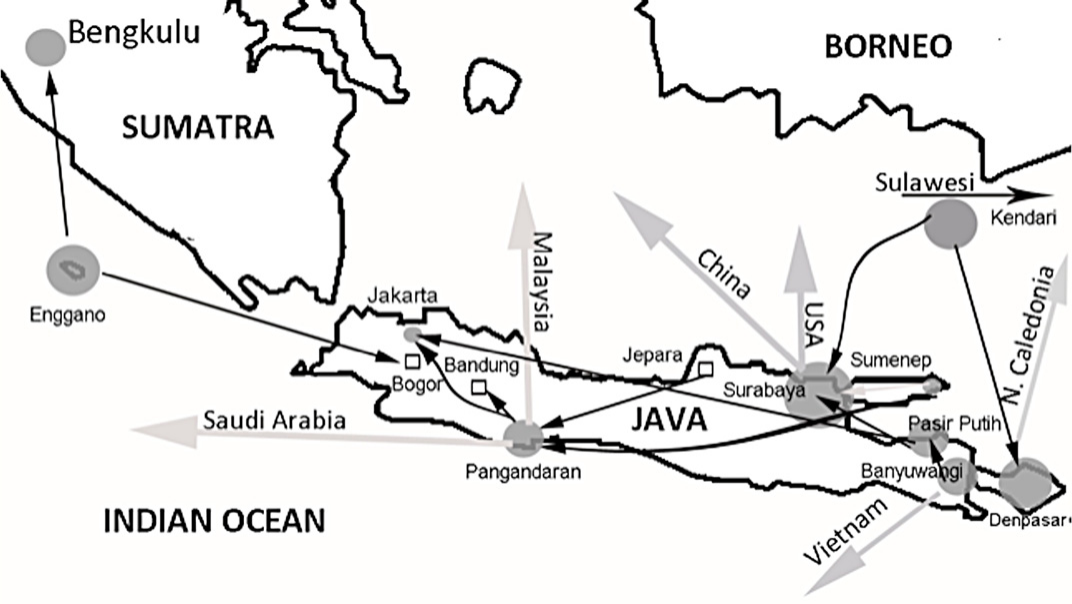
Shell trade networks. Protected marine molluscs shell trade networks in Java and Bali, Indonesia. Size of circles are proportional to the volume of trade: small = 10s, medium-100s, large-1000s and very large = 10,000s. Kendari is situated in south-central Sulawesi 800 km northeast of Bali.

In terms of trade networks and similarities in species composition of protected molluscs observed in trade or confiscated by the authorities (Fig 3), clear links occur between Pangandaran and Surabaya (Baroni-Urbani Buser coefficient of 0.913) and Sumenep (0.837) and between Pasir Putih and Surabaya (0.853) and Banyuwangi (0.840). Surabaya emerges as a centre for the trade in protected mollusc shells with clear links to Sumenep on nearby Madura (0.913) and Kendari on Sulawesi (0.913). Likewise, links between Bali and Sulwesi (0.775) are evident. At an international level, trade links between these Indonesian islands and North America occur as well as the Asia-Pacific region.

### 5. Monetary value

Prices in Pangandaran and Pasir Putih differed greatly between species, and were largely determined by the size of the shell, their (perceived) rarity, origin (with shells that were brought in from elsewhere being more expensive) and quality (damaged specimens being cheaper; shells with more intricate patterns commanding higher prices, as did some particularly nicely shaped individuals). It is worth noting that none of the traders mentioned the species’ protected status or inclusion on Appendix II of CITES as a reason to increase the asking price.

**Fig 4:**
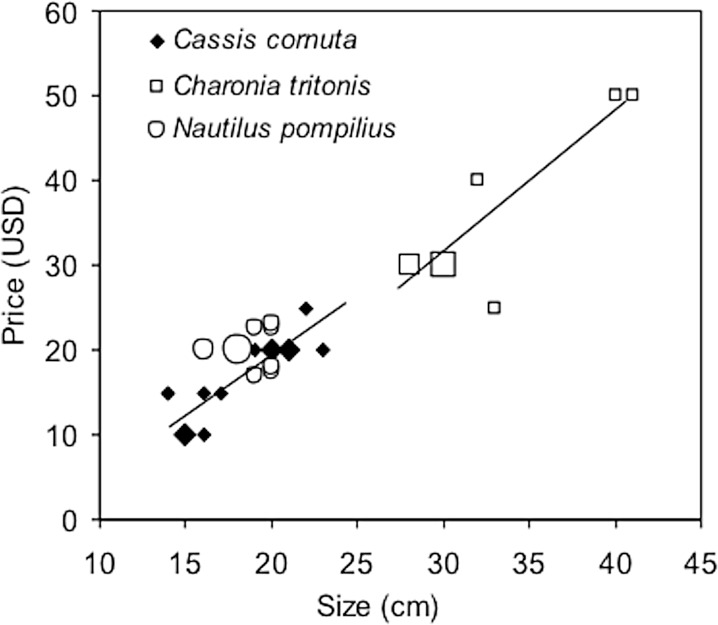
Price-size relationships for shells in trade. Prices of horned helmet *Cassis cornuta*, Triton’s trumpet *Charonia tritonis* and chambered nautilus *Nautilus pompilius* shells in relation to size, in Pangandaran, Indonesia, June 2013. Prices were given in Indonesian rupiah and are here converted to the US dollar at an exchange rate of 10,000 rupiah to the dollar. Small-sized symbols indicate single shells, medium-sized indicate two shells and large-sized symbols three shells; regression lines are for horned helmet and Triton’s trumpet only.

Asking prices for bear paw clam, China clam, marbled turban and commercial top shell at Pangadaran were typically between USD 0.15 to USD 0.30 per shell, with some quotes as high as USD 1.00. These shells are often sold in larger quantities and traders do lower their asking price when bartering for the purchase of multiple individuals. No prices were requested for these species from Pasir Putih, but it is likely that they fall in the same range. For chambered nautilus there appeared to be no difference between prices in Pangandaran or Pasir Putih, with shells averaging around USD 17.00. For shells measuring between 16 and 21 cm there was no effect of size on the asking price (Fig 4); instead pricing was based on the quality of the specimen and its pattern. The average price of horned helmet at Pangandaran was also around USD 17.00. Corrected for size, prices in Pasir Putih were typically 20% lower. Prices of Triton’s trumpet shells at Pangandaran averaged USD 35.00, and there was an increase of USD 2.00 for each cm increase in size. Prices at Pasir Putih appeared to be similar. Giant clam were sold for around USD 25.00, with lower prices for single shells and higher prices for complete double-shelled specimens. False trumpet was the most expensive shell ranging between USD 50.00 and USD 70.00. Details of prices and sizes are presented in Fig 4 and Table 3.

**Table 3:** Prices of shells. Prices (in USD) of large marine mollusc shells observed in trade in Pangandaran and Pasir Putih, Java. For species where there is a price-size relationship the shell size for the lowest and highest prices is included.

Species	Mean ±s.d. (n)	Minimum	Maximum	Notes
Chambered nautilus *Nautilus pompilius*	16.94±6.82 (9)	13.00	24.00	No price-size relationship
Horned helmet *Cassis cornuta*	16.92±4.80 (13)	15 cm, 10.00	22 cm, 25.00	Positive price-size relationship
Triton’s trumpet *Charonia tritonis*	35.00± 9.35 (9)	28 cm, 30.00	41cm, 50.00	Positive price-size relationship
False trumpet *Syrinx aruanus*	56.67±11.55 (3)	50.00	70.00	No price-size relationship
Giant clam	25.00±11.00 (7)	15.00	45.00	No price-size relationship
*Tridacna* spp				

Based on average prices and numbers observed, the retail value of the shells highlighted in Table 1 in Pangandaran totals between USD 3,500–5,200 (January) or USD 3,200–4,500 (June) and that of Pasir Putih between USD 3,100–4,800. This excludes the smaller protected species that are traded in larger volumes but at lower prices per individual shells (with asking prices of less than USD 0.50 these may add a few hundred dollars to the total, at most). In monetary terms the most valuable species was the horned helmet with estimated retail values of between USD 1,500 and USD 2,200 per visit. Using asking prices from Pangandaran and Pasir Putih as a guide, the total value of the shells confiscated in the last 8 years, as summarised above, totals over USD 700,000. In order of decreasing monetary importance the species are horned helmet (USD 500,000), chambered nautilus (USD 60,000), Triton’s trumpet (USD 55,000) and giant clam and false trumpet (both USD 45,000).

## Discussion

We found an open trade in marine mollusc shells protected under Indonesian law in two of the largest open markets in Java, with hundreds to thousands of individual shells for sale. Trade in protected species did not differ from trade in non-protected species (i.e. all were displayed openly and asking prices were, according to vendors, not influenced by the species’ protected status). The trade at these two markets appeared to cater largely for Indonesian tourists and only to a small degree for an international clientele. In recent years only a limited number of surveys actually quantifying levels of trade have been published on the marine mollusc shell curio trade. Gössling et al. [3] surveying the island of Zanzibar, Tanzania, estimated annual turn-overs of 724 shells for horned helmet, 1036 for Triton’s trumpet, and 1428 giant clams, amongst others; from their paper it is however not clear how many of these were actually observed in trade. Asking prices of these shells on Zanzibar fell typically in the lower range of what was requested in Pangandaran, noting however, that their survey [3] took place in 2002 (i.e. 11 years prior to our survey, and prices may be more comparable at present). Floren [35] reported on the marine mollusc shell trade in Mactan, the Philippines, documenting a substantial trade in protected marine molluscs, similar to what we observed in the present study. Species protected under Philippine law that were frequently offered for trade included horned helmet (estimated monthly turn-over of 5,000–7,000 shells), Triton’s trumpet, giant clam and chambered nautilus. Dias et al. [8] assessed the trade in marine shells in northeastern Brazil, observing no less than 116 species for sale, but did not quantify the volume of trade nor reported prices of individual shells. Gibbons and Remaneva [7] surveyed the marine mollusc shell trade in southeastern Madagascar and recorded amongst others Triton’s trumpet, horned helmet and giant clam for sale. The numbers of these species actually observed in trade in Madagascar were small, in the order of 10 to 20 shells per species. Finally, Nijman and Nekaris [36] reported on the trade in protected marine mollusc shells as part of a larger wildlife trade survey in Bali, and observed 94 chambered nautilus, 11 horned helmet and 3 Triton’s trumpet for sale.

We found little evidence of temporal differences in the availability of shells in Pangandaran and data from the two most commonly confiscated species (horned helmet and chambered nautilus) do not indicate temporal patterns in trade, or at least, confiscations happen throughout most months of the year. It is possible that harvesting does follow a more seasonal pattern.

The data compiled during this study on the numbers of confiscated marine molluscs in Java and Bali hint at a substantial and parallel international trade in these species. Data from nautilus imports into the United States [37] (see below) and wholesale companies in Indonesia offering large quantities of protected marine mollusc shells for sale strengthen this assertion. As such there may be need for additional international trade regulations, possibly as part of CITES [38]. Currently only giant clam are included on Appendix II of CITES and while each year at least several giant clams originating from Indonesia are confiscated in other countries, these numbers are dwarfed by the number of non-CITES listed, but locally legally protected species that are illegally shipped out of Indonesia.

In 1994, based on reports that there had been a reduction in the quantity and quality of shells available for sale in local shell shops, Australia put forward a proposal to have Triton’s trumpet included on Appendix II of CITES regulating all international trade [39], but withdrew it prior to the Conference of Parties because of lack of quantitative trade data. The rationale behind the proposal was that (i) the continued considerable but unmonitored and uncontrolled trade interest in Triton’s trumpet; (ii) the vulnerability of the species to over-collection; (iii) its role as a predator of high trophic status on coral reefs, and particularly its role in controlling Crown of thorn starfish (*Acanthaster planci*); and (iv) the lack of knowledge of the species’ habits and life history, made it appropriate for the species to be included on Appendix II of CITES [39]. Triton’s trumpets are collected from coral reefs throughout their range with relative ease because of their large size (up to 45 cm), accessibility and striking shell markings. The availability of SCUBA apparatus, outboard motors and cheap fuel had facilitated the collection of large numbers of molluscs, including Triton’s trumpet.

While generally little is known about the international trade in marine mollusc shells within and from Indonesia based on information from Indonesia itself, some data are available from importing countries. DeAngelis [37] reported that for the period 2005–2010, the USA alone imported over 95,000 whole nautilus shells, nearly all wild-caught, mainly from the Philippines and Indonesia. The import from Indonesia totalled some 74,000 items and close to 2,700 kg of nautilus-derived products (including whole shells, jewellery and shell products). Details on the species traded are unknown but chambered nautilus is the most common nautilid in trade in Indonesia suggesting a significant illegal international trade in the species. Floren (2003: 48–49) [35] reported that the exploitation of chambered nautilus in the Philippines falls under the Fisheries Administrative Order 168 of 1990 requiring permits to collect and export this species. Currently, the US Fish and Wildlife Service and TRAFFIC, the wildlife trade monitoring network, are involved in research to gain a better understanding of the status and the impact of nautilus fishing and trade on wild populations [40] (C.R. Shepherd, pers. comm.). Proposals are also in preparation for the inclusion of chambered nautilus on Appendix II of CITES [37].

### Legality of the trade

The trade we report on here appears to be largely illegal under Indonesian law. Legally protected species are displayed openly; neither the local government that operate these facilities, nor officers from the regional offices of Natural Resources Conservation Agency, officers from the Department of Forestry, nor the police seem to enforce wildlife protection legislation at these sites [41]. Of the twelve marine molluscs included in Indonesia’s list of protected species only the commercial topshell can be traded legally, provided they have been collected in designated areas following the official procedures in volumes up to the allocated quotas. Given the illegality of the trade in the protected species, it is perhaps not too farfetched to surmise that most the topshell trade may not be legal; at the very least it would be difficult to ascertain whether the shells in trade were actually fished in designated areas. Unfortunately the Indonesian authorities are not forthcoming with these quota figures, other than those involving CITES listed species, which are published annually on the CITES website. Giant clams can be traded commercially, provided they are derived from mariculture. No trade in wild-caught giant clam is permitted. Yet CITES trade data indicate that a total of 110 shells were exported between 2005 and 2012, most of which had been confiscated. While false trumpet are not included in the list of protected species, trade in them is only allowed following a quota system and no quota for the species has been allocated. As such trade in false trumpets should not be allowed.

There appears to be a great disparity in the number of raids conducted in the different parts of the country. In the city of Surabaya enforcement seems to be significantly higher than in other parts of the country. The realization that over the last 8 years only 32 protected shells have been confiscated in one operation in the Jakarta area–a very important centre for the export of marine products [42]- points to a lack of enforcement effort in the capital. Equally, we expect levels of trade to be significantly higher in the coastal towns of Java (Cirebon, Semarang), Bali (Sanur, Singaraja) and Madura (Sumenep, Bangkalan) as well as the major ports in Sumatra (e.g. Medan) and Sulawesi (e.g. Makassar). While we were able to document links to an eclectic mix of countries such as the USA, Saudi Arabia, Malaysia, Vietnam, China and New Caledonia, it is clear that the real international network linking countries to the Indonesian marine mollusc trade must be more substantial.

While a number of confiscations have been made involving 1000s or even 10,000s of shells from wholesalers or exporters, the median number of shells confiscated in the 18 raids in the period 2005–2013 listed above is less than 80 shells. It is noteworthy that none of the known and reported seizures were made in Pangandaran or Pasir Putih, despite the openness of the trade and the importance of these markets in trade. Likewise, reports of exports of wild-caught giant clam, the confiscations of illegally imported giant clam, and the operation of wholesale exporters all suggest there is a clear discrepancy between state laws and regulation, and on-the-ground enforcement. It appears that in Indonesia curbing the large-scale trade in marine molluscs is not on the radar of enforcement agencies; as such the distinction between illegal, illicit and licit is blurred.

It is perhaps worth noting that even to those involved in monitoring the trade there is a clear hiatus in the awareness of the legality of the trade. For instance, DeAnglis [37] expressed that there were concerns about the legality of trade (‘ban’) in chambered nautilus in Indonesia. She noted that “Information on this ban is conflicting [], and the basis, nature, specificity, and duration of a ban have yet to be confirmed. Recent U.S. trade data, communications with experts, and information obtained from web-based commercial traders indicate that trade in shells and jewellery of Indonesian origin is on-going” [37] (p8). In the period DeAnglis conducted her research (2005–2010), chambered nautilus had been on the Indonesian list of legally protected species for over two decades, but clearly this information had not been adequately communicated to importers or other interested parties. Indeed the openness of the trade, both physically on the ground within Indonesia and by consulting wholesale traders’ Internet sites, suggests nothing but a legal basis for this trade to the uninitiated observer.

## Recommendations

On the basis of our study it is clear that there is scope for improvement of real on-the-ground protection of marine molluscs in Indonesia, at the local, regional, national and international level.

The open, illegal sale and consistent presence of protected species in markets that should be easy to monitor points at a clear neglect of duties by Indonesian wildlife conservation authorities and the local government agencies that operate at Pangandaran and Pasir Putih [41]. Wildlife protection laws are not being enforced as intended and suggest a lack of pressure on the authorities to treat these illegal sales as a priority. Collectors, middlemen, traders and consumers (both domestic and international) engaged in illegal activities must be held accountable for their actions and prosecuted, and law enforcers must be given incentives to carry out their duties in this regard with greater efficiency.

The unequal distribution of seizures in different parts of the country, with most of the raids being executed by the agencies in Bali and Surabaya, may reflect the geographical pattern of trade in marine mollusc shells, but in all likelihood also results from differential enforcement efforts. Based on the size of the ports and the volumes of trade in other marine animals, we expect higher levels of illegal trade in the major seaports of Medan, Jakarta and Makassar. It is imperative that enforcement efforts are stepped up to control trade in these areas.

The false trumpet is not included in the list of protected species [11]. Its restricted distribution, large size (it being the largest extant gastropod), slow life history, high volumes observed in trade, and the high price it commands at markets (possibly indicating rarity) suggests a need for legal protection equivalent to other large marine molluscs in Indonesia. The species is available in the wholesale market suggesting that international trade could indeed pose a threat to its survival. It is also clear that at least some of the law enforcement agencies do consider it protected as witnessed by the confiscation of 768 false trumpet shells in Surabaya [33]. Given the similarities in appearance between crusty and chambered nautilus (crusty nautilus has a more pronounced umbilicus, i.e. the depressed central area of the shell [43]) serious consideration should be given to including this species on the list of protected species (in fact, had it been known in 1987 that the species occurred in Indonesian waters it may well have been included in the initial list of 12 protected species). As with CITES listings, the inclusion of a species on the protected species list in itself may not lead to increased protection -the data presented here are evidence of that- but it may be a first step towards curbing over-exploitation [44] [45].

Large volumes of legally protected marine molluscs from Indonesia are imported into the USA [37]. There is no *a priori* reason to assume that similar imports are absent from for instance the EU, Japan or China. We feel that at least part of the responsibility of adherence to wildlife protection laws lie with the importing countries. We firstly urge the EU and other major wildlife importers to invest in a monitoring system similar to LEMIS in the USA allowing one to assess these imports and secondly for these importers to check the legality of their shipments [38]. Conversely, we urge the Indonesian authorities to be more active with informing importing countries about trade restrictions (including bans and quotas).

While the data from Java and neighbouring islands comprise just one dataset, in recent years it has become clear that at least a number of the larger marine molluscs currently not included on the appendices of CITES are traded internationally in large volumes. These include chambered nautilus [37] [44] [45], Triton’s trumpet [39] and horned helmet [35] (this study). A detailed assessment of the international trade in nautilids is underway in anticipation of future CITES proposals [40] and similar assessment of other large molluscs may be warranted. While inclusion onto the appendices of CITES in itself does not guarantee improved management or better enforcement of existing national laws, it does draw attention to the issue of over-exploitation of these animals, and ensures that quantitative data are available on trade patterns and trends, possibly leading to improved protection.

## Methods

### 1. Market surveys

VN and KAIN visited Pangandaran on the south coast of West Java for three days each in January and June 2013 and Pasir Putih on the north coast of East Java for one day in June 2013, whereas DS visited Pangandaran for three days in August 2013. Based on previous visits made by VN in the 1990s and 2000s these two localities were identified as two of the largest open markets for marine mollusc shells in western Indonesia. Both Pangandaran and Pasir Putih fall under local government management (Ciamis and Situbondo regencies, respectively) and are operated by a state enterprise of the local authority. Visitors are required to purchase an entry ticket (Pangandaran: USD 0.25 per visitor plus USD 1.42–4.02 for private vehicles, the amount depending on the size of the car; Pasir Putih: USD 1.00 per visitor and USD 1.00 for a private car) and traders pay an annual fee to the local government operators.

All survey days covered weekends and coincided with Indonesian public holidays, with more shops / stalls selling marine curios than at other times. All shops selling marine curios were visited. We made detailed accounts for a selected number of species that, because of their slow life histories, protected status within Indonesia, and / or numbers observed may be negatively affected by being traded in Pangandaran and / or Pasir Putih (Table 4). For all species whole individuals were counted, but for giant clam, vendors often offer halves for sale, and these were counted as one. When vendors displayed complete giant clams these were counted as one as well. Large shells were mostly offered as whole unworked specimens. A small number may have been included in for instance mosaics or other handicrafts but as they would have been difficult to identify these were not included in the current survey.

**Table 4:** Conservation status of marine molluscs. The IUCN Red List status of marine molluscs traded in Java and Bali, Indonesia, with information on their maximum sizes and age of reproductive maturity. Key: n/a = not assessed, LR/cd = Lower Risk, conservation dependent, VU = Vulnerable

Species	IUCN	Range	Maximum size	Reproductive maturity	Reference
Chambered nautilus *Nautilus pompilius*	n/a	Indo-Pacific	20 cm	12–15 years	[46]
Crusty nautilus *Allonautilus scrobiculatus*	n/a	Pacific	21 cm	12–15 years	[47]
Horned helmet *Cassis cornuta*	n/a	Indo-Pacific	31 cm	-	[48]
Triton’s trumpet *Charonia tritonis*	n/a	Indo-Pacific	40 cm	-	[49]
False trumpet *Syrinx aruanus*	n/a	Indo-Pacific	91 cm	-	[50]
Bear paw clam *Hippopus hippopus*	LR/cd	Asia-Pacific	40 cm	5 years	[51, 52]
Giant clam *Tridacna gigas*	VU	Indo-Pacific	130 cm	8–10 years	[53]

For selected species, prices were requested from dealers (depending on the species this ranged from two to sixteen traders), and we here report the initial quotes: prices would have gone down probably by at least 20 to 30% after bargaining or if more than one item had been purchased at a time. Prices are here converted to US dollar using an exchange rate of IDR 10 000 to USD 1. More so in Pangandaran than in Pasir Putih informal conversations were held with traders (in Bahasa Indonesia, Indonesia’s national language) as to glean information about origins, turn-over, buyers, and the legality of the trade (for sample questions see S1 File). Over the two years some 30 traders and wholesalers were active in Pangandaran and with almost all of them we discussed the marine mollusc trade. Some were more forthcoming and knowledgeable and gave detailed accounts of the trade whereas others were less open or had less insight into the trade. For species that were offered for sale by only a restricted number of traders, such as false trumpet or crusty nautilus, the information is derived from these traders only. The trade was open, there was no need to resort to undercover techniques, no traders were informed about the nature of our survey, and no wildlife was purchased. We asked the traders for information but they themselves were not the subjects of the research (i.e. they were not asked about their own views, attitudes, concerns, behaviour or anything else pertaining to them as individuals) and as such we did not require ethics clearance from our university research ethics committee. Market surveys were part of a larger conservation research programme that included wildlife market surveys in West and East Java (RISTEK research permit 11/TKPIPA/FRP/SM/XI/2013 to DS and KAIN, 039/SIP/FRP/SM/II/2012 to Johanna Eva Rode and KAIN). After the surveys were conducted we communicated our findings to the Director of Biodiversity Conservation of the Directorate General of Forest Protection and Nature Conservation.

### 2. Assessment of illegal trade in and from Java and Bali

We searched for Indonesian wholesale traders that offered protected marine molluscs shells for sale; two of them, both in Pangandaran, were visited, for others we obtained information from their websites. In December 2013 we conducted a search for documents reporting the illegal trade in protected shells in Indonesia (search terms included the Indonesian names of the species (e.g. *kerang kima*, *kerang kepala kambing*) in combination with the words seizure or confiscation (root: *serah*, *sita*), the acronyms of the agencies that do most of the confiscations (BKSDA, PHKA) or customs (*bea cukai*); to locate exporters we used the |ndonesian or Latin names of the species in combination with the words export or *ekspor*). We restricted ourselves to the islands of Java and Madura (administratively under the province of East Java) and their close neighbour Bali, but we included data from other islands when there was a clear link with Java or Bali (for instance shipments from or to Java). The websites of the regional offices of Natural Resources Conservation Agency (BKSDA), Department of Forestry (Departemen Kehutanan, responsible for implementation of protected species laws), Department of Fisheries (Departemen Kelautan dan Perikanan) and the Directorate General of Customs (Directorate Jendral Bea Cukai) were searched for documents relating to the export or confiscation of protected shells. In addition, we searched the websites of the major Indonesian newspapers and periodicals (Kompas, Surabaya Tribun, Bali Post, Jurnal Nasional, Tempo) for articles related to trade in protected marine molluscs. We furthermore downloaded data for all *Tridacna* and *Hippopus* exported from Indonesia from the CITES trade database (www.cites.org) for the period 2005–2013 (data from 2014 were not yet available), and include transactions of wild-caught individuals (source code W—wild) and confiscations or illegal transactions (source code I–illegal).

### 3. Analysis

Prices of marine mollusc shells typically increase as one progresses further up the trade chain. In Indonesia, horned helmet shells are typically bought from collectors for less than USD 0.50, sold on to middlemen for one-and-a-half to twice that amount, and are offered for sale at resorts for a minimum of USD 7.50 (Pasir Putih) to USD 9.00 (Pangandaran). These are similar to prices at Indonesian classified ads websites, but significantly lower than prices paid for on the international markets, where asking prices for single horned helmet shells of USD 65.00 to USD 100.00 are not uncommon (some of this difference in price is justified to cover freight and other costs involved in transport). We used quotes from Pangandaran to arrive at an estimate of the gross retail value of the marine mollusc shells in trade. For some, but not all, species there is a clear relationship between price and size. In case where we had information on sizes we used this to estimate values; when these were not available we used average values as observed in Pangandaran and Pasir Putih.

We compared differences and similarities between surveys, sites and regions with chi-square tests (using Yates’ correction where appropriate) and Baroni-Urbani Buser coefficient that takes into account total numbers of species observed at two sites, the number of shared species between sites and the number of shared absences [54]. Significance was accepted when P<0.05 in a two-tailed test.

## Supporting Information

S1 File Examples of questions asked to obtain insight in the trade of marine molluscs in Java and Bali. (DOCX)
