# Supplementary material for: Large-Scale Trade in Legally Protected Marine Mollusc Shells from Java and Bali, Indonesia
Source: PLoS One. 2015 Dec 30;10(12):e0140593. doi: 10.1371/journal.pone.0140593 (PMC4696778; doi:10.1371/journal.pone.0140593)
Supplement: S1 File — (DOCX) [file pone.0140593.s001.docx]

**S1 File. Examples of questions asked to obtain insight in the trade of marine molluscs in Java and Bali**

**To the people at the gate:**

“How much does it cost to enter?”, “How many people visit Pangandaran / Pasir Putih each year?” “How many of them are foreigners?”

**To the traders:**

“What is this?”, “What name do you give to this type of shell?” “What species is it?”, “Is this a different type than this one?”

“Where does it come from?” “Is it collected on the beach?” “Is it brought in by fishermen?”

“Who are the main buyers?” “Where do they come from?”

“How much does this one cost?”, “and this one?”, “Why is this one more expensive than that one?”

The interviews were open ended, and questions varied in their precise wording.
